# Cerebellar Theta-Burst Stimulation Impairs Memory Consolidation in Eyeblink Classical Conditioning

**DOI:** 10.1155/2018/6856475

**Published:** 2018-10-09

**Authors:** Jessica Monaco, Lorenzo Rocchi, Francesca Ginatempo, Egidio D'Angelo, John C. Rothwell

**Affiliations:** ^1^Brain Connectivity Center, C. Mondino National Neurological Institute, Pavia, Italy; ^2^Department of Brain and Behavioral Sciences, University of Pavia, Pavia, Italy; ^3^Sobell Department of Motor Neuroscience and Movement Disorders, UCL Institute of Neurology, London, UK; ^4^Department of Biomedical Sciences, University of Sassari, Sassari, Italy

## Abstract

Associative learning of sensorimotor contingences, as it occurs in eyeblink classical conditioning (EBCC), is known to involve the cerebellum, but its mechanism remains controversial. EBCC involves a sequence of learning processes which are thought to occur in the cerebellar cortex and deep cerebellar nuclei. Recently, the extinction phase of EBCC has been shown to be modulated after one week by cerebellar continuous theta-burst stimulation (cTBS). Here, we asked whether cerebellar cTBS could affect retention and reacquisition of conditioned responses (CRs) tested immediately after conditioning. We also investigated a possible lateralized cerebellar control of EBCC by applying cTBS on both the right and left cerebellar hemispheres. Both right and left cerebellar cTBSs induced a statistically significant impairment in retention and new acquisition of conditioned responses (CRs), the disruption effect being marginally more effective when the left cerebellar hemisphere was stimulated. These data support a model in which cTBS impairs retention and reacquisition of CR in the cerebellum, possibly by interfering with the transfer of memory to the deep cerebellar nuclei.

## 1. Introduction

The cerebellum is a brain region involved in different neural processes, including fine motor control, sensorimotor learning, and motor memory consolidation [1, 2]. The eyeblink classical conditioning (EBCC) is one of the most studied paradigms to investigate cerebellar mechanisms underlying associative motor learning in healthy [3, 4] and pathological [5–8] conditions. Learning, timing, and prediction are the primitive functionalities of the cerebellum, and EBCC, despite its simple execution, can capture them all [9]. The learning process underlying EBCC is made of fast events such as acquisition and extinction, likely taking place in the cerebellar cortex, while a slower consolidation phase is probably linked to the activity in the deep cerebellar nuclei (DCN) [2, 4].

In humans, it is possible to modulate cerebellar activity through repetitive transcranial magnetic stimulation (rTMS) [10–14]. Among rTMS protocols, continuous theta-burst stimulation (cTBS) is a fast patterned form of rTMS known to induce synaptic plasticity through a long-term depression-like mechanism [15]. Cerebellar cTBS has been used to modulate EBCC only in few studies. In a first work, Hoffland and coworkers [16] demonstrated that cTBS, when applied on the right cerebellar hemisphere before EBCC, was able to interfere with the acquisition of conditioned responses (CRs) tested just after conditioning, although retention, reacquisition, and extinction of CR was not affected after one week. By contrast, Monaco and colleagues [17] demonstrated that right cerebellar cTBS applied after EBCC led to an impairment of the extinction phase, but not reacquisition of CR, again after one week. The latter finding was interpreted by postulating that cTBS interfered with a fast learning process, represented by the extinction phase, while consolidation, related to a slower learning process, was left untouched. However, the long time interval between the two EBCC sessions did not allow clarification of the effect which was due to an impairment of the early transfer phase from the cerebellar cortex to the DCN nuclei or depended on a disruption of plasticity in the latter.

It could be possible that the effect on retention and reacquisition in these studies was lost because of the long interval between the experimental sessions; thus, with this study, we primarily aimed to clarify the timing of cTBS interference with the mentioned fast learning process. To investigate this aspect, we tested EBCC in healthy subjects 5 minutes after cTBS was applied to the cerebellar hemispheres.

The asymmetry of several cerebellar functions is known [18], but no lateralization of EBCC has been reported; therefore, as a second aim, we investigate this issue, then cTBS was applied on the left and right cerebellar hemispheres in separate groups.

## 2. Materials and Methods

### 2.1. Subjects

Thirty-six right-handed healthy subjects (20 males, mean age: 28.3 ± 4.1, see Table 1 for further details) participated in this study. An informed written consent was obtained from all subjects; the experimental procedure was approved by the local ethical committee and conducted in accordance with the Declaration of Helsinki. All subjects were naïve to EBCC and had no history of neurological, psychiatric, or hearing disorders; they were not taking drugs that are active at the central nervous system level. Subjects were randomly divided into three groups of 12 subjects: “right cTBS (r-cTBS),” “left cTBS (l-cTBS),” and “sham cTBS (s-cTBS)”. EBCC was tested as previously explained [17], before (T0) and 5 minutes after (T1) cTBS.

### 2.2. Electromyographic (EMG) Recordings

EMG signals were recorded (D360 amplifier; Digitimer Ltd., Welwyn Garden City, UK) using 9 mm diameter Ag-AgCl surface cup electrodes, placed over the target muscle. Eyeblink was recorded bilaterally from the orbicularis oculi (OO) muscle, with the recording electrode placed over the lower lid and the reference electrode 2 cm far from the lateral cantus.

Motor-evoked potentials (MEPs) were recorded from the first dorsal interosseous (FDI) muscle of the right hand. The recording electrode was placed over the FDI and the reference electrode on the first metacarpophalangeal joint. EMG was amplified, band-pass filtered (5 Hz to 2 kHz) and sampled (5 kHz) using CED 1401 power analog-to-digital converter (Cambridge Electronic Design, UK). Data were recorded and analyzed using Signal 5.02 software (Cambridge Electronic Design, UK).

### 2.3. Eyeblink Classical Conditioning Protocol

The conditioned stimulus (CS) was a loud (70–80 dB; 2000 Hz), with tone lasting 400 ms, and delivered via binaural headphones. The CS inconsistently produced an acoustic startle response (“alpha blink”) occurring within 200 ms after the CS [19]. The unconditioned stimulus (US) was a square electrical pulse of 200 *μ*s length and an intensity equal to five times the somatosensory threshold (ST), delivered over the right supraorbital nerve 400 ms after the CS. Pairs of CS and US at 400 ms interstimulus interval (ISI) were delivered in 6 acquisition blocks (each consisting of 9 CS-US pairs, 1 US-only, and 1 CS-only trial). A seventh block consisted of 11 CS-only trials to measure extinction. The intertrial interval was randomised between 10 and 30 seconds to reduce habituation.

### 2.4. Transcranial Magnetic Stimulation

cTBS was delivered using a Magstim Super Rapid magnetic stimulator (Magstim, UKTM), connected to a 70 mm figure-of-eight coil. cTBS, consisting of three-pulse bursts at 50 Hz repeated every 200 ms given in a continuous train lasting 40 s (600 pulses), was delivered at 80% of the active motor threshold (AMT), i.e., the lowest intensity-evoking five MEPs of at least 200 *μ*V in 5 of 10 consecutive trials while subjects maintained a low level tonic contraction (10–15% of maximal voluntary contraction) in the right FDI muscle. Activation of the finger was required only to measure the AMT, whereas for the rest of the experiment, the FDI muscle was kept at rest. cTBS was applied over the lateral cerebellum, at a point 1 cm inferior and 3 cm lateral to the inion, with the coil handle pointing superiorly, putatively targeting the posterior lobe of the lateral cerebellum [20, 21]. Sham stimulation was delivered with the same intensity as that used in the cTBS protocol but with the coil held perpendicularly to the scalp over the cerebellar midline in order to produce an ineffective cortical activation [21].

### 2.5. Data Analysis and Statistics

EMG bursts were considered “alpha blinks,” i.e., nonconditioned responses inconsistently produced by the CS, if their amplitude exceeded 50 *μ*V and their latency was <200 ms after the CS, whereas they were regarded as CR if latency was 200–400 ms after the CS (ending before the US). For the CS-only trials, EMG bursts occurring 200–600 ms after the CS were considered CRs [22]. Before undergoing ANOVA procedures, normal distribution of data was assessed by means of Shapiro–Wilk's test. Since most variables were not normally distributed, both nontransformed and after exponential and logarithmic transformations, nonparametric tests were mostly used. To ensure that there were no differences in the three groups in terms of age and stimulation parameters, three different one-way between-group ANOVAs were used to compare AMT, ST, and age in the group studied. Three different dependent Friedman's ANOVAs, one for each group, were performed on the number of CRs before cTBS to assess whether the learning process was effective, i.e., the number of CRs increased after block 1. This was done under the hypothesis of an effective baseline learning of CR, as reported before [8, 17]. Given that the EBCC protocol was the same in the three groups, we expected that baseline acquisition of CR would not be different. To confirm this, several Kruskal–Wallis tests were performed to compare EBCC in the three groups across all learning blocks before cTBS was delivered. To investigate the effect of cTBS on EBCC, the number of CRs before cTBS was subtracted from the number of CRs after cTBS and the difference (number of CRs in EBCCpost − number of CRs in EBCCpre, ∆CR) was used as a variable, with the prediction that real stimulation would impair acquisition of CR. As a global measure of cTBS effect on learning, we first used a Kruskal–Wallis test to investigate the effect of cTBS on the sum of ∆CRs across all blocks (blocks 1 to 7) in the three groups. Secondly, to assess the effect of cTBS on EBCC in the three groups in detail, several Kruskal–Wallis tests were performed in the number of CRs in each block. We finally tested the effects of cTBS on retention and extinction. To assess this first, a Kruskal–Wallis test comparing the ∆CRs between block 6 before cTBS and block 1 after cTBS across the three groups was performed. To evaluate the effect of cTBS on extinction, two Kruskal–Wallis tests were performed on CRs in block 7 across the three groups, before and after cTBS. The level of statistical significance was preset at *p* < 0.05. Mann–Whitney test or Wilcoxon signed-rank test was used for post hoc comparisons when the main tests disclosed any significant *p* values. Unless otherwise stated, all results are indicated as mean ± standard error (SEM).

## 3. Results

The experiments were well tolerated by all subjects, and no adverse effects were observed.

The three ANOVAs performed to investigate the differences in age and stimulation parameters (ST, AMT) and age across the three groups did not show significant main effects (Table 1).

### 3.1. Baseline Learning

**Figure 1:**
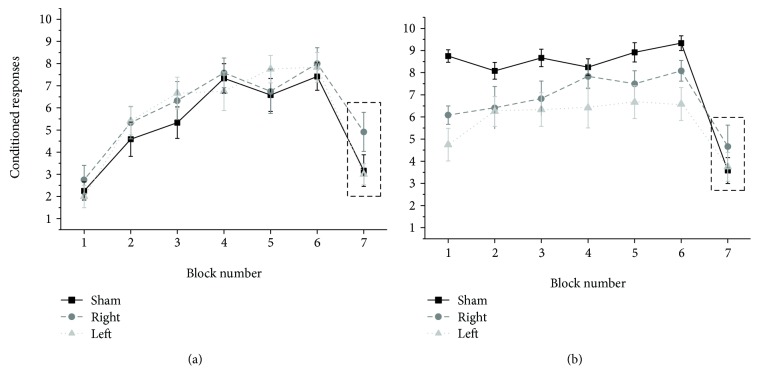
EBCC learning, extinction, and consolidation in the right, left cTBS, and sham groups at T0 (a) and at T1 (b). In all groups, the number of CRs was significantly higher in blocks 2–6 than in block 1 (all *p* < 0.05), whereas differences in CRs in each block were not statistically significant among the three groups. After cerebellar conditioning, both the right and left cerebellar cTBSs led to a decrease in the number of CRs in blocks 1–6 compared to sham stimulation. Dotted rectangles indicate the extinction block. Error bars indicate the standard error.

The three Friedman's ANOVAs performed to compare the number of CRs in block 1 with other blocks showed significant *p* values for all cTBS groups (right: *χ*^2^ = 41.548, *p* < 0.001, left: *χ*^2^ = 47.121, *p* < 0.001, and sham: *χ*^2^ = 47.009, *p* < 0.001). Post hoc analyses showed that the number of CRs in block 1 was significantly lower than that in blocks 2 to 6 (all *p* < 0.05) in all groups, thus confirming an effective learning process (Figure 1). The Kruskal–Wallis tests on the number of CRs at T0 in the three different groups did not disclose any significant difference in CR across all the blocks, thus confirming that the baseline learning process was similar (all *p* > 0.05) (Figure 1).

### 3.2. Effects of cTBS on CR Reacquisition

**Figure 2:**
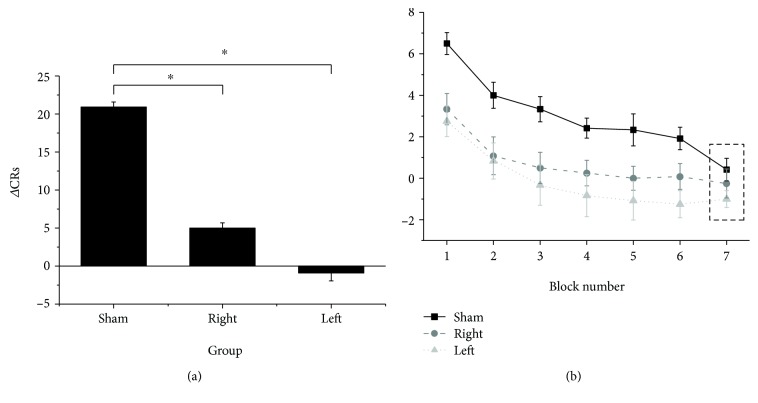
Effects of cTBS on ∆CR, measured as the number of CRs after cTBS minus the number of CRs before cTBS. (a) Both the right and left cTBS groups induced a statistically significant decrease in the total number of ∆CRs compared to the sham group (both *p* < 0.001). (b) ∆CR was significantly smaller in the right and left cTBS groups compared to the sham group in blocks 1–6 (all *p* < 0.05). Dotted rectangles indicate the extinction block. Error bars indicate the standard error. Asterisks indicate the statistical significance.

The effect of cTBS was different based on the protocol used. At T1, in the sham group, CR restarted immediately at the same level that was attained at T0 (about 80%). Conversely, in the right and left cTBS groups, CR restarted at a level significantly lower than that at T0 (about 60%, and 50%, respectively) and remained below the control level all along T1. This was demonstrated by the Kruskal–Wallis test performed on the sum of ∆CRs across all blocks, which showed a significant difference across the three groups (H2 = 16.777, *p* < 0.001). Mann–Whitney tests showed that ∆CRs were significantly lower both in the right and left cerebellar cTBS groups than in the sham cTBS group, respectively (*U* = 10.5, *p* < 0.001 and *U* = 14, *p* = 0.001 (Figure 2(a)). A more detailed result was provided by the Kruskal–Wallis tests performed on each block separately, which showed a significant difference in ∆CRs in the three groups across acquisition blocks 1 to 6 (all *p* < 0.05) while ∆CRs in extinction block 7 was similar (H2 = 2.723, *p* = 0.256). Follow-up Mann–Whitney tests confirmed that the decrease of ∆CRs was significant across acquisition blocks 1 to 6 both in the case of the right and left cerebellar cTBSs (all *p* < 0.05) (Figure 2(b)). Although cTBS applied to the left cerebellar hemisphere was slightly more effective than that applied to the right cerebellar cTBS, the difference between the two groups is not significant (all *p* > 0.05).

### 3.3. Effects of cTBS on Retention and Extinction

**Figure 3:**
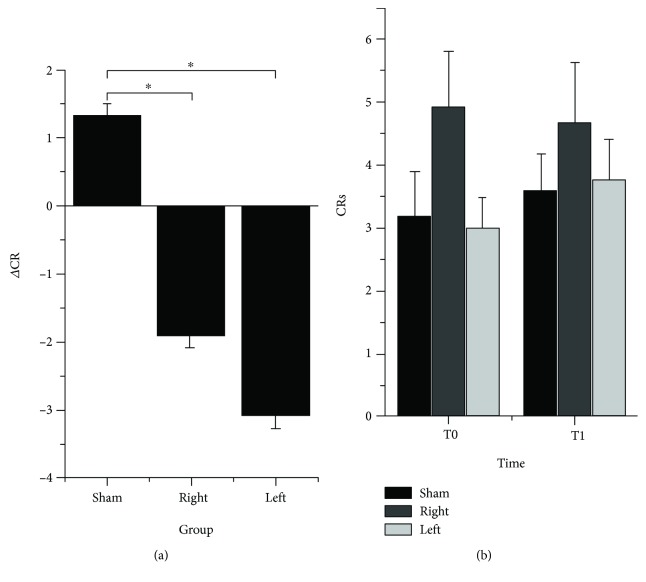
Effects of cTBS on retention and extinction. (a) Effects of cTBS on retention, measured as the difference of CRs in block 1 after cTBS and CRs in block 6 before cTBS separately in each group. Both the right and left cTBS groups led to a statistically significant decrease in retention compared to the sham group (both *p* < 0.001). (b) Effects of cTBS on extinction, measured as the number of CRs in block 7 before (T0) and after (T1) cTBS in the three groups separately. There were no statistically significant effects of cTBS on extinction. Error bars indicate the standard error. Asterisks indicate the statistical significance.

Retention showed a significant decrease (H2 = 17.625, *p* < 0.001), and post hoc analyses showed a higher reduction in the right cTBS group and left cTBS group (*p* < 0.001 for both) than in the sham cTBS group (Figure 3(a)). The difference between the effects on retention of the left and right cTBSs was not significant (*p* = 0.093).

Extinction showed no significant difference across the three groups, both before (H2 = 0.969, *p* = 0.325) and after cTBS (H2 = 0.376, *p* = 0.540), and also, no statistically significant difference was noted when the number of CRs in block 7 was compared in T0 and T1 in each group separately (Figure 3(b)) (all *p* > 0.05).

## 4. Discussion

Overall, the present results showed that cTBS delivered a few minutes after EBCC can interfere with EBCC retention and reacquisition tested soon thereafter, but leaving extinction unaffected. These results suggest the ability of cTBS to disrupt a fast phase of cerebellar associative learning, likely mediated by the cerebellar cortex.

### 4.1. Effects of cTBS on Reacquisition of Conditioned Responses

The observed decrease in the reacquisition of CR is in agreement with the study of Hoffland and coworkers [16], with a small caveat related to the fact that in that study, cerebellar cTBS was applied just before, instead of after, EBCC. We speculate that cTBS can have a similar effect when applied just before or just after EBCC, respectively, by decreasing the excitability of the cerebellar cortex, and thus hampering the formation of a new motor memory, or by interfering with the encoding of a newly formed memory pattern.

The effects observed in the present study are also in agreement with those of cathodal transcranial direct current stimulation (TDCS). The latter, applied on the right cerebellar hemisphere, was able to decrease acquisition of CR [23]. Considering that both cTBS and cathodal TDCS are noninvasive brain stimulation techniques that are able to decrease neural excitability, this finding confirm our hypothesis that the decrease in the number of CRs observed here is caused by a disruption of activity in the cerebellar cortex.

It is already known that cTBS can induce changes in neuronal excitability, possibly through LTD-like effects on synapses [15]. This has been confirmed in different physiological and behavioral outcomes when applied on a range of cortical areas and on the cerebellum both in healthy subjects and pathologic conditions [24–28]. Given these findings, a likely possibility is that our results are due to an impairment in the activity of the cerebellar cortex. In particular, the electrical activity induced by cTBS might have interfered with plasticity generated by EBCC pairing [29] and, eventually, altered Purkinje cell activity, which is considered crucial in the expression of conditional blink responses [30].

The cerebellar cortex is not the only site where plastic changes happen during EBCC. Several experimental, theoretical, and robotic experiments have revealed that EBCC requires distributed plasticity mechanisms involving multiple synaptic sites both in the cerebellar cortex and DCN [31–33]. It has been hypothesized that EBCC involves a two-phase learning process with a fast phase occurring in the cortex and a slower phase occurring primarily in the DCN [17, 34, 35]. The process evolves dynamically, so that plasticity is rapidly acquired in the cortex and then progressively transferred to the deeper structures. Thus, one can argue that cTBS has interrupted this memory transfer and therefore impaired memory consolidation. This might have happened by altering the expression of cortical plasticity at the numerous sites at which it is normally developed [31, 36, 37].

### 4.2. Effects of cTBS on Retention of Conditioned Responses

An interesting question is why in both the previous studies by Monaco and colleagues [17] and Hoffland and coworkers [16] there was no decrease in retention of CR one week after cerebellar cTBS. One possibility is that the memory trace substantially involved the cerebellar cortex, but it is more resistant to the effects of cTBS compared to the acquisition on CR. This would be in line with the finding that acquisition is more sensitive to a range of interventions compared to retention; the latter can be made resistant by overtraining [38] and that extinguished CR can spontaneously reappear with the passage of time [39–41]. Alternatively, it is possible that long-term storage of memory for EBCC is more dependent on extracerebellar structures, which are less accessible to the effects of cTBS. Although there is debate about the roles of cerebellar cortex and DCN in the acquisition and retention of EBCC [3, 29], the second hypothesis would be supported by data indicating that retention of associative memory mostly relies on activity in the DCN [42–44], particularly in their synapses with afferent mossy fibers [45–49].

### 4.3. Effects of cTBS on Extinction of Conditioned Responses

Another important point is that in the previous work by Monaco and colleagues [17], but not in the one by Hoffland and coworkers [16], extinction was selectively impaired one week after cerebellar cTBS, whereas in the present paper, extinction was not changed immediately after cTBS. Again, as for reacquisition and retention, we can only speculate about putative mechanisms due to the noninvasive nature of our investigation. To explain the mentioned discrepancy, one possibility is to hypothesize that the neural bases of extinction differ from those underlying acquisition. Indeed, it was suggested that whereas reversible cerebellar cortex and nucleus interpositus are essential for acquisition and retention of CR [50, 51], extinction is more dependent on inhibitory transmission from the interpositus to the inferior olive and from activity in the latter structure [52, 53]. Although the inferior olive is quite distant from our site of cTBS, remote secondary effect have been described after repetitive TMS [54–57]. Thus, we can speculate that the result on extinction seen in the work of Monaco and colleagues [17] was caused by secondary remote effects of cTBS on the inferior olive. The long latency of the observed effects might be justified considering that, in addition to inducing changes in electrical activity of neurons and synaptic transmission, repetitive TMS has been demonstrated to influence slow biological processes such as receptor trafficking and synthesis of neurotrophic factors [58].

### 4.4. Lateralization of cTBS Effects

Our results are seemingly at odds with the notion, which is supported by data obtained both in animal models and in humans [59, 60], that cerebellar lesions impair CR acquisition only when EBCC is tested in the ipsilateral eye. The effects of cTBS might be not as spatially specific as those of discrete lesions due to the current spread; it is thus possible that cTBS delivered on the left cerebellar hemisphere had an effect on the contralateral hemisphere as well.

## 5. Conclusions

Overall, the present findings suggest that cTBS can impair memory consolidation processes in the cerebellum, possibly by interfering with memory transfer from the cerebellar cortex to the DCN.

## Figures and Tables

**Table 1:** Mean ± SEM values of age and stimuli parameters in the right cTBS, left cTBS, and sham cTBS groups. *p* values are relative to the main effect of factor “group” in the ANOVA.

	Type of stimulation				
	Right cTBS	Left cTBS	Sham cTBS	*F*	*p*
Age (years)	28.3 ± 4.25	28.6 ± 3.8	27.9 ± 4.7	0.093	0.912
ST (mA)	2.3 ± 0.4	2.4 ± 0.8	2.3 ± 0.8	0.088	0.916
AMT (% MSO)	45.4 ± 6.9	44.9 ± 8.4	43.6 ± 9.7	0.151	0.860

mA = milliamperes; AMT = active motor threshold; MSO = maximal stimulator output.

## Data Availability

The data used to support the findings of this study are available from the corresponding author upon request.

## Abbreviations

AMT: Active motor threshold
CRs: Conditioned responses
CS: Conditioned stimulus
DCN: Deep cerebellar nuclei
EBCC: Eyeblink classical conditioning
EMG: Electromyography
FDI: First dorsal interosseous
ISI: Interstimulus interval
MEPs: Motor evoked potentials
TBS: Theta-burst stimulation
TMS: Transcranial magnetic stimulation
ST: Somatosensory threshold
US: Unconditioned stimulus.
